# Supplementary material for: Differential pathogenic and commensal response of Staphylococcus aureus and Staphylococcus epidermidis toward chemical signals of human skin
Source: J Bacteriol. 2026 Apr 22;208(5):e00119-26. doi: 10.1128/jb.00119-26 (PMC13192271; doi:10.1128/jb.00119-26)
Supplement: Supplemental tables and figures — Tables S1 to S4 and Figures S1 to S6. [file jb.00119-26-s0001.pdf]

Table S1. Minimum inhibitory concentration (MIC) of palmitoleic acid (C16:1Δ9).

	**Media**	**MIC (μM)**
S. aureus USA300	TSB (glu), Unbuffered	75
	TSB (NG), Unbuffered	100
	TSB (NG), pH 5.5	400
S. epidermidis M23864:W2	TSB (glu), Unbuffered	200
	TSB (NG), Unbuffered	200
	TSB (NG), pH 5.5	>1600

Abbreviations: TSB (glu)= tryptic soy broth with glucose; TSB(NG)= tryptic soy broth without glucose

Table S2. Strains and plasmids used in this study.

Strain or Plasmid:		Description:	Reference:
S. aureus:			
1	USA300 LAC	Community associated MRSA: wild type strain cured of resistance plasmids	(1)
2	RN4220	r_K^- m_K^+; capable of accepting foreign DNA	(2)
3	USA300Δ*graS*	USA300 with markerless *graS* deletion	(3)
4	USA300Δ*graR*	USA300 with markerless *graR* deletion	This study
S. epidermidis:			
5	M23864:W2	*S. epidermidis* wild type stain, characterized by the Human Microbiome Project, obtained from human skin	BEI resources HM-144
6	LK401	Commensal strain recovered from human skin	(4)
7	LK346	Commensal strain recovered from human skin	(4)
8	LK345	Commensal strain recovered from human skin	(4)
9	LK354	Commensal strain recovered from human skin	(4)
10	LK460	Commensal strain recovered from human skin	(4)
11	LK470	Commensal strain recovered from human skin	(4)
12	LK1136	Commensal strain recovered from human skin	(4)
13	LK257	Commensal strain recovered from human skin	(4)
E. coli:			

14	DH5α	F⁻ Φ80*lacZ*ΔM15 *recA1 endA1 gyrA96 thi-1 hsdR17* ($r_K^-$$mK^+$) *supE44 relA1 deoR* Δ(*lacZYAargF*)*U169 phoA*	Invitrogen
**Plasmids:**			
15	pALC2073	Shuttle vector used for expression of genes under control of tetracycline-inducible Pxyl/tetO promoter in *S. aureus*; genes are expressed at a basal level in absence of induction	(5)
16	p*graS (SA)*	Promoterless *graS* gene of USA300 under transcriptional control of Pxyl/tetO promoter of pALC2073	(3)
17	p*graR* (SA)	Promoterless *graR* gene of USA300 under transcriptional control of Pxyl/tetO promoter of pALC2073	This study
18	p*graS* (SE)	Promoterless *graS* gene of *S. epidermidis* M23864:W2 under transcriptional control of Pxyl/tetO promoter of pALC2073	This study
19	p*graR* (SE)	Promoterless *graR* gene of *S. epidermidis* M23864:W2 under transcriptional control of Pxyl/tetO promoter of pALC2073	This study
20	pGYLux	*E. coli-S. aureus* shuttle vector harboring promoterless *luxABCDE* operon; Amp^r Cm^r	(6)

21	pGY*sspA::lux*	pGYLux with the promoter of *sspA* cloned upstream of *luxABCDE*	This study
22	pGY*esp::lux*	pGYLux with the promoter of *esp* cloned upstream of *luxABCDE*	This study

Table S3. Oligonucleotides used in this study.

Oligonucleotide:	Description:
graR-UP-SacII	AAGAGACCGCGGAGTATTTGCATCCATATCACC
graR-UP-attB1	ggggacaagtttgtacaaaaaagcaggctTGGGCCATAAAAAGCCTCC
graR-DW-SacII	GTAGGAAAAGGATATATCCGCGGTGAATAATTTGAAATGGGTAGC
graR-DW-attB2	ggggaccactttgtacaagaaagctcggtATCAATCTGACAGTTGTCCCC
graR-DEL-UP	TCATCACTTTCAGCAACGAAG
graR-DEL-DW	CCATAATAGCAATAAACTCGCC
sspA-p-F	GGAGGATCCTAGATGAAAGGTAAATTTTTAAAAGTTAG
sspA-p-R	CAAACAGTCGACACCTAAGATTCAAAAAGGC
esp-p-F	AATTGGCAGACGCGCGGGATCCAAAATCCCGTTC
esp-p-R	GTATTTACCATTGTCGACGTTGCTAACGC
graR (SE)-F	GCATTATCAAGGTACCATTCAATCAGGTGAGAAG
graR (SE)-R	CACTTTCAACTGAGCTCTCATAATCTATGTAGGCAACACC
graR (SA)-F	TTTGGTACCTGGCTTTGAAGTTGACTGCC
graR (SA)-R	TTTGGTACCCACAGGTGTTTTTATGTCGTGC
graS (SE)-F	AATGGTACCACCAAAGTTGTTGGTAAAGACTAC
graS (SE)-R	AATGGTACCCACTCCTGTCATACTTCAACC
pALC-F	TTAAACCTTCGATTCCGACC

pALC-R	GAGAAAATACCGCATCAGG
pGYlux-F	CTGTTGTTTGTCGGTGAACGT
pGYlux-R	ATTGGGGAGGTTGGTATGTAAGC

*Underlined nucleotides represent restriction digestion sites. Added attB1 and attB2 sites are indicated with lower cases font.

Table S4: Composition of Laurdan Buffer (7, 8).

SN	Reagents	Working concentration
1.	Sodium chloride (NaCl)	137mM
2.	Potassium chloride (KCl)	2.7mM
3.	Na_2HPO_4	10mM
4.	KH_2PO_4	1.8mM
5.	Glucose	0.2%
6.	DMSO	1%

REFERENCES

1. Arsic B, Zhu Y, Heinrichs DE, McGavin MJ. 2012. Induction of the Staphylococcal Proteolytic Cascade by Antimicrobial Fatty Acids in Community Acquired Methicillin Resistant Staphylococcus aureus. PLoS One 7:e45952.

2. Novick RP. 1991. Genetic systems in Staphylococci. Methods Enzymol 204:587–636.

3. Flannagan RS, Kuiack RC, McGavin MJ, Heinrichs DE. 2018. Staphylococcus aureus Uses the GraXRS Regulatory System To Sense and Adapt to the Acidified Phagolysosome in Macrophages. mBio 9:e01143-18.

4. Salamzade R, Cheong JZA, Sandstrom S, Swaney MH, Stubbendieck RM, Starr NL, Currie CR, Singh AM, Kalan LR. 2023. Evolutionary investigations of the biosynthetic diversity in the skin microbiome using lsaBGC. Microb Genom 9:mgen000988.

5. Bateman BT, Donegan NP, Jarry TM, Palma M, Cheung AL. 2001. Evaluation of a Tetracycline-Inducible Promoter in Staphylococcus aureus In Vitro and In Vivo and Its Application in Demonstrating the Role of sigB in Microcolony Formation. Infect Immun 69:7851–7857.

6. Mesak LR, Yim G, Davies J. 2009. Improved lux reporters for use in Staphylococcus aureus. Plasmid 61:182–187.

7. Wenzel M, Vischer N, Strahl H, Hamoen L. 2018. Assessing Membrane Fluidity and Visualizing Fluid Membrane Domains in Bacteria Using Fluorescent Membrane Dyes. Bio Protoc 8:e3063.

8. Mercier R, Domínguez-Cuevas P, Errington J. 2012. Crucial Role for Membrane Fluidity in Proliferation of Primitive Cells. Cell Rep 1:417–423.

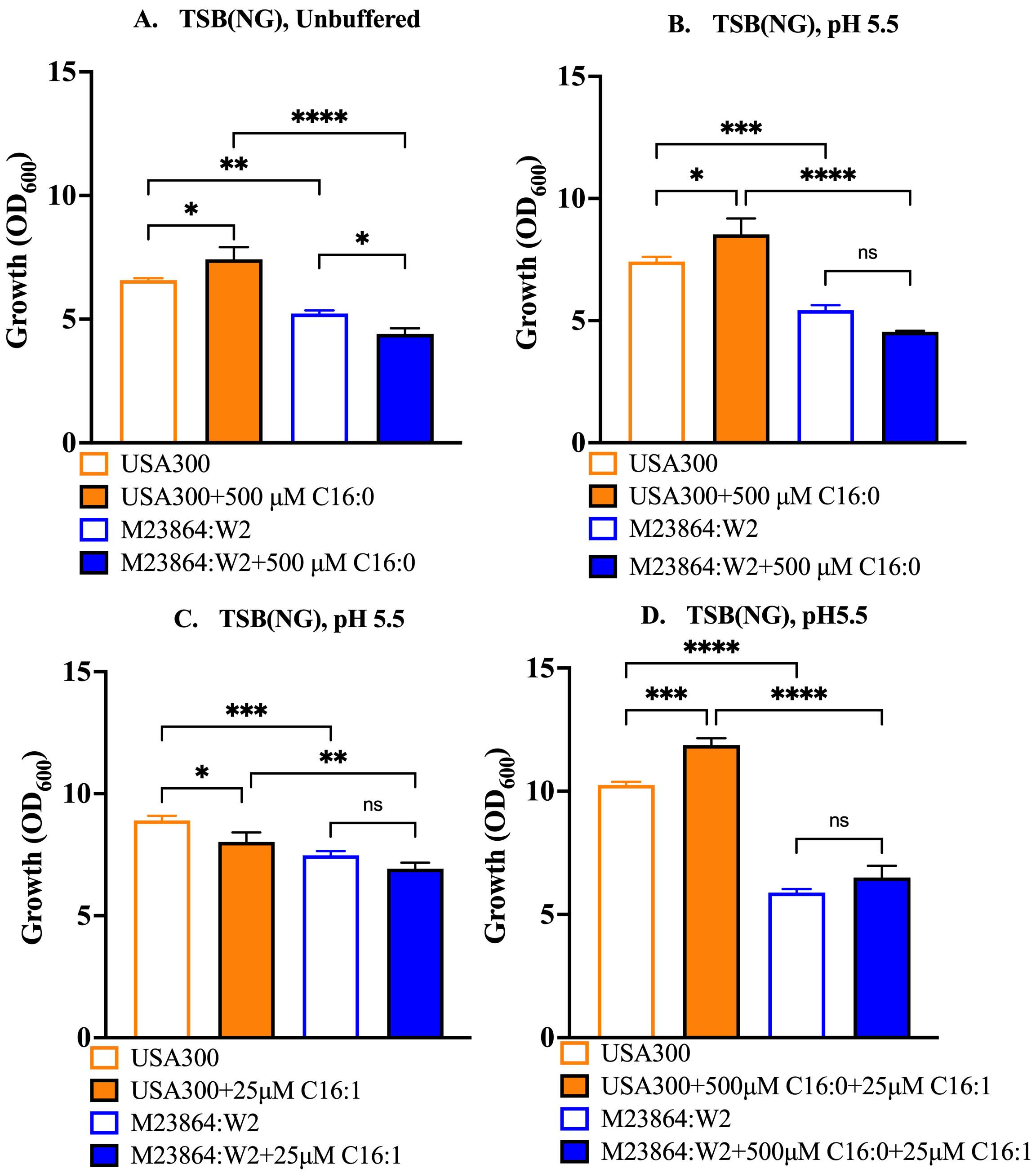

Fig. S1: Effect of acidic pH on the growth of *S. aureus* USA300 and *S. epidermidis* M23864:W2 in the presence of palmitic acid (C16:0), palmitoleic acid (C16:1), or both. Growth was assessed in triplicate flasks of TSB without glucose [TSB(NG)], either unbuffered and supplemented with C16:0 at indicated concentrations (A) or buffered to pH 5.5 and supplemented with C16:0 (B), C16:1 (C), or both C16:0 and C16:1 (D) and growth (OD_{600}) was measured at 24 hours. Each data represent mean ± SEM from triplicate flasks. Statistical significance was measured using One-Way ANOVA with Tukey's multiple comparisons test, ns= not significant, *p<0.05, *p<0.01, ***p<0.001, ***p < 0.0001.

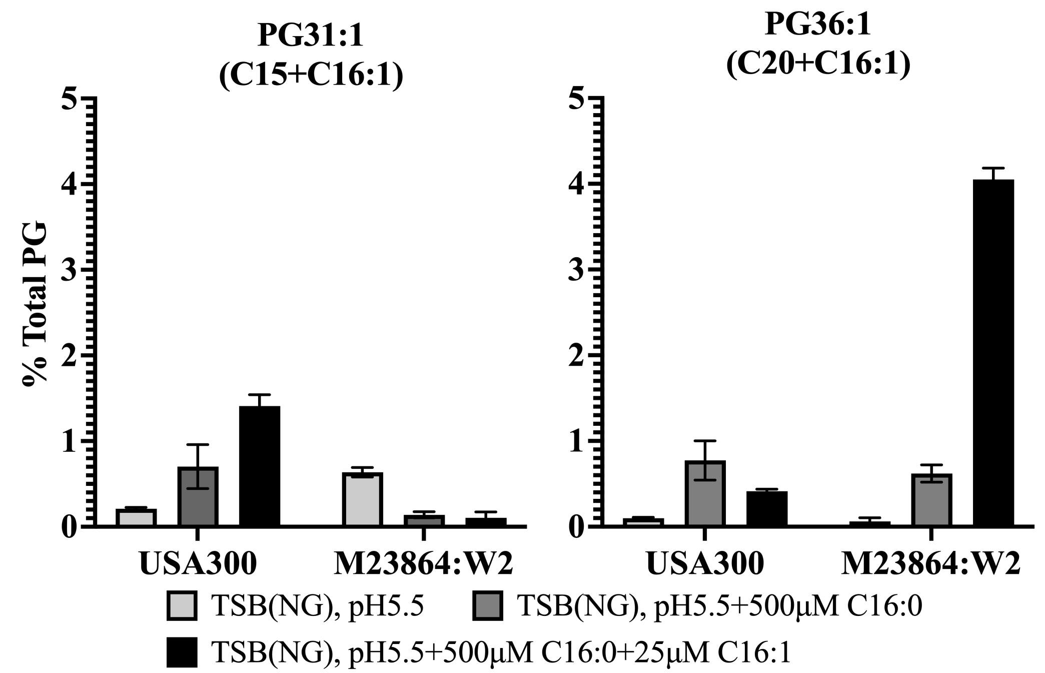

Fig. S2: Exogenous fatty acids are differentially incorporated into the phospholipid membrane of *S. aureus* USA300 and *S. epidermidis* M23864:W2. USA300 and M23864:W2 were grown in triplicate cultures to an OD600 of 0.6 in different media conditions; TSB(NG) pH5.5 alone and/or with palmitic acid (C16:0) or with both palmitic and palmitoleic acid (C16:1). Bacteria were then centrifuged and collected the sample pellets. Samples were homogenized, and lipids were extracted and analyzed through liquid chromatography–mass spectrometry at the Wayne State Lipidomics Core Facility. Results are presented as mean ± SEM of the percentage of total PG each lipid species represents. Minor PG species measured in cultures, PG31:1 (C15+C16:1) and PG36:1 (C20+C16:1). Each bar represent mean ± SEM from three biological replicates.

Q2FZL2 Glutamyl endopeptidase OS=Staphylococcus aureus (strain NCTC 8325 / PS 47) OX=93061 GN=sspA PE=1 SV=1 (36325.176 Da)

```
MKGKFLKVSS LFVATLTTAT LVSSPAANAL SSKAMDNHPQ QTQSSKQQTP KIQKGGNLKP  60
LEQREHANVI LPNNDRHQIT DTTNGHYAPV TYIQVEAPTG TFIASGVVVG KDTLLTNKHV 120
VDATHGDPHA LKAFPSAINQ DNYPNGGFTA EQITKYSGEG DLAIVKFSPN EQNKHIGEVV 180
KPATMSNNAE TQVNQNITVT GYPGDKPVAT MWESKGKITY LKGEAMQYDL STTGGNSGSP 240
VFNEKNEVIG IHWGGVPNEF NGAVFINENV RNFLKQNIED IHFANDDQPN NPDNPDNPNN 300
PDNPNNPDEP NNPDNPNNPD NPDNGDNNNS DNPDAA
```

Q5HN75 Glutamyl endopeptidase OS=Staphylococcus epidermidis (strain ATCC 35984 / DSM 28319 / BCRC 17069 / CCUG 31568 / BM 3577 / RP62A) OX=176279 GN=gseA PE=3 SV=1 (30830.988 Da)

```
MKKRFLSICT MTIAALATTT MVNTSYAKTD TESHNHSSLG TENKNVLDIN SSSHNIKPSQ  60
NKSYPSVILP NNNRHQIFNT TQGHYDAVSF IYIPIHGGYM SGSGVVVGEN EILTNKHVVN 120
GAKGNPRNIS VHPSAKNEND YPNGKFVGQE IIPYPGNSDL AILRVSPNEH NQHIGQVVKP 180
ATISSNTDTR INENITVTGY PGDKPLATMW ESVGKVVYIG GEELRYDLST VGGNSGSPVF 240
NGKNQVIGIH YGGVDNKYNS SVYINDFVQQ FLRNNIPDIN IQ
```

Fig. S3: Peptide sequence comparison of MS-identified protein bands with reference glutamyl endopeptidases from *S. aureus* (SspA) and *S. epidermidis* (Esp). Protein bands excised from SDS-PAGE were subjected to mass spectrometry (MS) analysis, and the resulting peptide sequences were mapped against the reference sequences of SspA from *S. aureus* and Esp from *S. epidermidis*. Highlighted regions represent peptide fragments detected by MS that matched the respective reference protease sequences. Matched peptides from the *S. aureus* band align with the SspA sequence (orange), while matched peptides from the *S. epidermidis* band align with the Esp sequence (purple). Conserved residues and matched peptide positions confirm the identity of the gel-purified proteins as SspA in *S. aureus* sample and Esp in *S. epidermidis* sample.

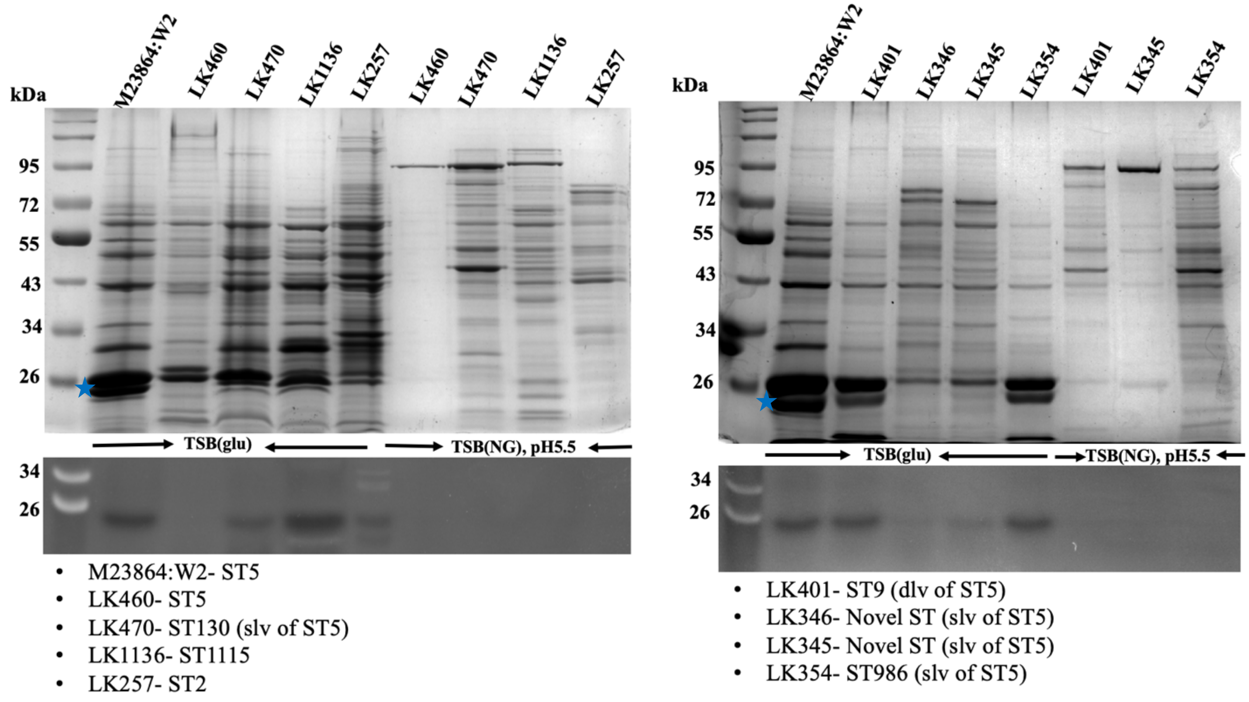

Fig. S4: Esp expression across diverse *S. epidermidis* strains under neutral and acidic pH conditions. Selected *S. epidermidis* strains were grown in TSB with glucose [TSB (glu)] at unbuffered condition or in TSB without glucose buffered to pH 5.5 [TSB(NG), pH5.5] and culture supernatant were collected after 20 hours. For SDS-PAGE profiles of secreted proteins (upper), TCA-precipitated proteins equivalent to 2.5 OD_{600} units of culture supernatant were applied to each lane, while for zymogram analyses (bottom), a volume of culture supernatant equivalent to 0.075 OD_{600} unit was applied to each lane. Stars indicate the position of signature proteins Esp serine protease (blue star).

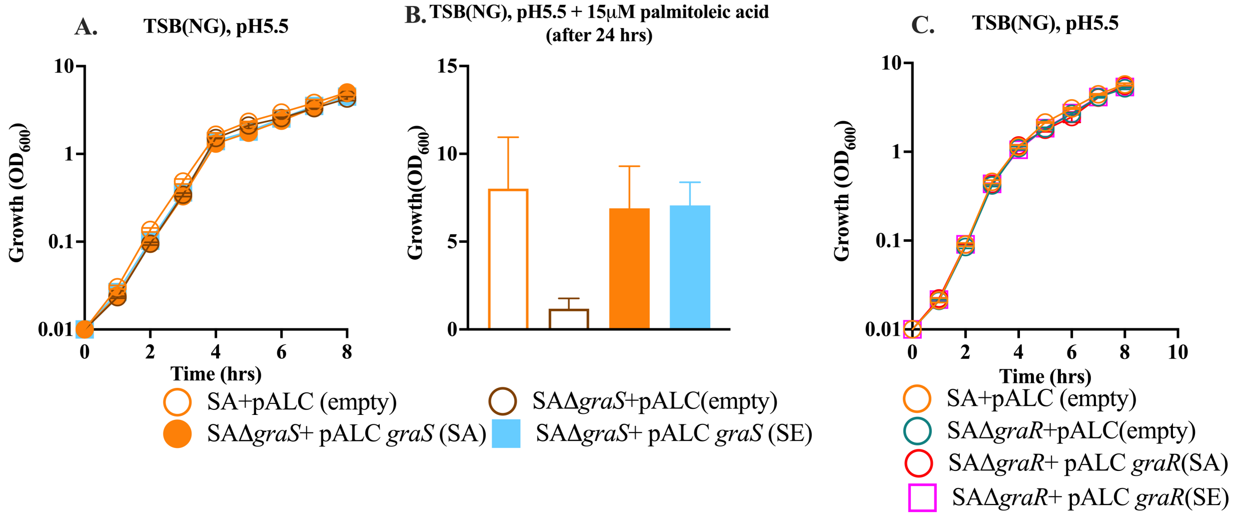

Fig. S5: Growth of *S. aureus* USA300 Δ*graS* and Δ*graR* mutants complemented with *graS* or *graR* from *S. aureus* or *S. epidermidis* under acidic conditions. (A, C) Growth of *S. aureus* USA300 Δ*graS* and Δ*graR* mutants complemented with *graS* or *graR* from *S. aureus* (SA) or *S. epidermidis* (SE) in TSB without glucose buffered pH 5.5. Cultures were inoculated at an initial OD_{600} of 0.01 and monitored hourly for 8 h. (B) Endpoint growth of the strains assessed after 24 h in TSB without glucose pH 5.5 supplemented with 15 µM palmitoleic acid. Data represent means ± SEM from three independent biological replicates.

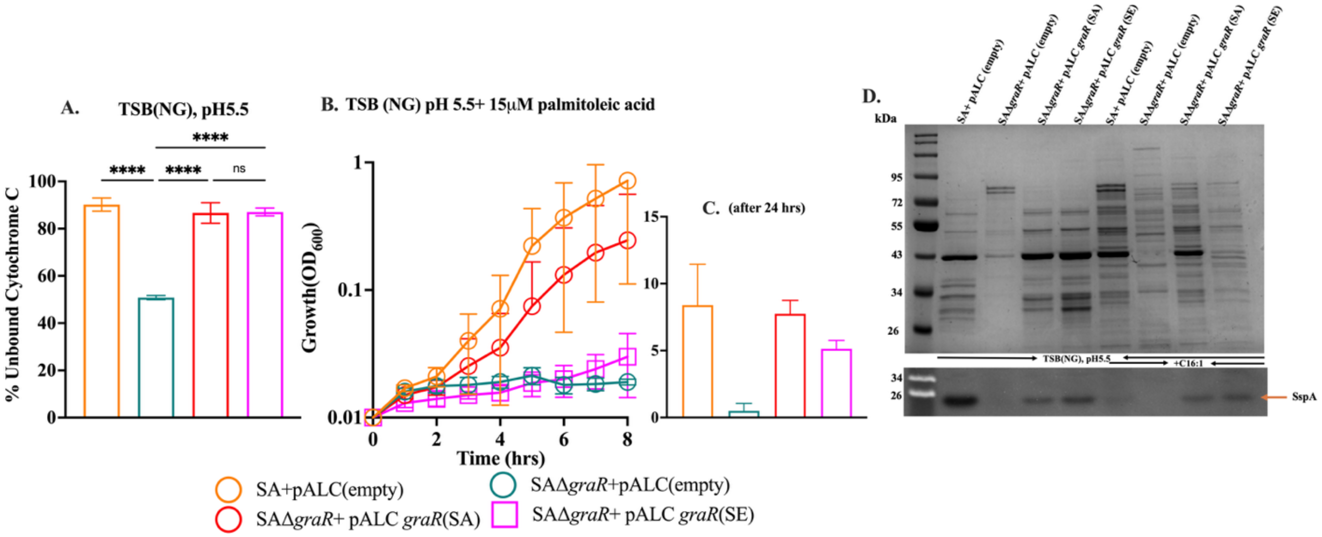

Fig. S6: *S. epidermidis* GraR restores growth, surface charge, and protease activity in the *S. aureus* Δ*graR* mutant under skin-like acidic pH and fatty acid conditions. Growth of USA300 wild type and Δ*graR* mutants carrying empty plasmid (pALC) or complemented with *graR* from *S. aureus* (SA) or *S. epidermidis* (SE) in TSB (NG) pH 5.5 supplemented with 15 µM palmitoleic acid. (A) Cultures were inoculated at $OD_{600} = 0.01$, and growth was monitored hourly for 8 h. (B) Endpoint growth of the same strains assessed after 24 h in TSB(NG) pH 5.5 supplemented with 15 µM palmitoleic acid. (C) SDS-PAGE (upper) and zymography (lower) of culture supernatants collected after 24 h from the indicated strains grown in TSB(NG) at pH 5.5 alone or with 15 µM palmitoleic acid. For SDS-PAGE, TCA-precipitated proteins equivalent to 2.5 OD_{600} units were loaded per lane; for zymography, a volume equivalent to 0.075 OD_{600} unit was loaded per lane. (D) Cytochrome c binding assay of the same strains grown in TSB(NG) at pH 5.5 (D). Data represent mean ± SEM from three independent experiments performed in triplicate. Statistical significance was determined by one-way ANOVA with Tukey's multiple comparisons test (*p<0.05, **p < 0.01, ****p < 0.0001, ns = not significant).
